# SARS-CoV-2 seroprevalence in healthcare workers at a frontline hospital in Tokyo

**DOI:** 10.1038/s41598-021-87688-9

**Published:** 2021-04-16

**Authors:** Hiroshi Fukuda, Kuniaki Seyama, Kanami Ito, Tomohiko Ai, Shuko Nojiri, Satoshi Hori, Mitsuru Wakita, Kaori Saito, Yuka Shida, Rie Nagura, Mayu Hasegawa, Chiaki Kanemoto, Mayumi Tokuhara, Katsunobu Okajima, Yukio Yoshikawa, Narimasa Katsuta, Takamasa Yamamoto, Mayumi Idei, Yuki Horiuchi, Kotoko Yamatani, Shigeki Misawa, Toshio Naito, Takashi Miida, Hiroyuki Sato, Nobutaka Hattori, Yoko Tabe, Kazuhisa Takahashi

**Affiliations:** 1 Department of General Medicine, Juntendo University Graduate School of Medicine, Bunkyo, Tokyo Japan; 2 Department of Respiratory Medicine, Juntendo University Graduate School of Medicine, Bunkyo, Tokyo Japan; 3 Department of Safety and Health Promotion, Juntendo University, Bunkyo, Tokyo Japan; 4 Department of Clinical Laboratory Medicine, Juntendo University Graduate School of Medicine, 2-1-1, Hongo, Bunkyo, Tokyo 113-8421 Japan; 5 Medical Technology Innovation Center, Juntendo University, Bunkyo, Tokyo Japan; 6 Infection Control Unit, Juntendo University Hospital, Bunkyo, Tokyo Japan; 7 Department of Infection Control Science, Juntendo University Graduate School of Medicine, Bunkyo, Tokyo Japan; 8 Department of Clinical Laboratory, Juntendo University Hospital, Bunkyo, Tokyo Japan; 9 Department of Neurology, Juntendo University Graduate School of Medicine, Bunkyo, Tokyo Japan; 10 Department of Next Generation Hematology Laboratory Medicine, Juntendo University Graduate School of Medicine, Bunkyo, Tokyo Japan

**Keywords:** Infectious diseases, Public health, Virology

## Abstract

Healthcare workers (HCWs) are highly exposed to severe acute respiratory syndrome coronavirus 2 (SARS-CoV-2) infection. The actual coronavirus disease (COVID-19) situation, especially in regions that are less affected, has not yet been determined. This study aimed to assess the seroprevalence of SARS-CoV-2 in HCWs working in a frontline hospital in Tokyo, Japan. In this cross-sectional observational study, screening was performed on consented HCWs, including medical, nursing, and other workers, as part of a mandatory health checkup. The screening test results and clinical characteristics of the participants were recorded. The antibody seroprevalence rate among the 4147 participants screened between July 6 and August 21, 2020, was 0.34% (14/4147). There was no significant difference in the seroprevalence rate between frontline HCWs with a high exposure risk and HCWs working in other settings with a low exposure risk. Of those seropositive for SARS-CoV-2, 64% (9/14) were not aware of any symptoms and had not previously been diagnosed with COVID-19. In conclusion, this study provides insights into the extent of infection and immune status in HCWs in Japan, which has a relatively low prevalence of COVID-19. Our findings aid in formulating public health policies to control virus spread in regions with low-intensity COVID-19.

## Introduction

Coronavirus disease (COVID-19) caused by severe acute respiratory syndrome coronavirus 2 (SARS-CoV-2) has evolved into a pandemic with sustained human-to-human transmission^1^. Clinical studies have found that approximately 5% of symptomatic patients develop severe symptoms and more than 80% show mild symptoms^2^ and that individuals with minimally symptomatic or asymptomatic infection carry the virus^3^.

Healthcare workers (HCWs) engage in the clinical care of patients with suspected and confirmed COVID-19 and are therefore exposed to a high risk of infection^4^. Therefore, HCWs working in regions severely affected by the COVID-19 pandemic have a high prevalence of SARS-CoV-2 infection detected by polymerase chain reaction^5^. Although clarifying the risk of COVID-19 among frontline HCWs is important for infection control, the actual situation, especially in regions that are less affected by COVID-19 has not yet been determined. Systematic screening for SARS-CoV-2 is an effective tool for the surveillance of the pandemic, and the seroprevalence of SARS-CoV-2 among HCWs who are most exposed to SARS-CoV-2 infection is an effective indicator of the spread of SARS-CoV-2 infection.

In this study, we aimed to determine the SARS-CoV-2 seroprevalence in HCWs working at a frontline hospital in the Tokyo area, in order to determine the prevalence of past infection, both symptomatic and asymptomatic, using a validated chemiluminescent assay^6^.

## Methods

### Study design and participants

This cross-sectional observational study was conducted between July 6 and August 21 as part of a mandatory health checkup of employees working at the Juntendo University Hospital and employees and students of the Juntendo University Graduate School of Medicine, Tokyo, Japan. A total of 4147 participants underwent antibody identification from blood specimens. All participants completed a web-based questionnaire on their medical history and health status. Detailed medical history interviews regarding possible SARS-CoV-2 infection were conducted by clinical interviewers to SARS-CoV-2 antibody-positive individuals. The participants were classified as follows: medical doctors (n = 1111), nurses (n = 1308), laboratory technicians (n = 236), paramedics (n = 314), administrative staff (n = 510), researchers (n = 632), and others (n = 36). To better classify the individual risk rate, three categories were identified. High-risk exposure (HR) occupation: frontline HCWs, including medical doctors and nurses. Medium-risk exposure (MR) occupation: non-frontline paramedics or laboratory personnel transporting or handling specimens from patients. Low-risk exposure (LR) occupation: administrative staff of the hospital, researchers, and others who may have minimal chance of exposure. All healthcare personnel who had any contact with infected/suspected cases of COVID-19 wore proper personal protective equipment recommended by the US Centers for Disease Control and Prevention (CDC) (https://www.cdc.gov/coronavirus/2019-ncov/hcp/using-ppe.html).

This research complied with all relevant national regulations and institutional policies, was conducted in accordance with the tenets of the Helsinki Declaration, and was approved by the Institutional Review Board (IRB) at Juntendo University (IRB #20–089). Informed consent was obtained from all the study participants.

### Measurement of SARS-CoV-2

We used the US Food and Drug Administration-approved Elecsys Anti-SARS-CoV-2 electrochemiluminescence immunoassay system (Roche Diagnostics, Basel, Switzerland), which is based on the modified double-antigen sandwich immunoassay with recombinant nucleocapsid protein (N) and measures SARS-CoV-2 total antibody (pan immunoglobulin) with a fully automated Cobas e801 analyzer (Roche Diagnostics) (https://www.accessdata.fda.gov/cdrh_docs/presentations/maf/maf3358-a001.pdf). According to the FDA, the Elecsys Anti-SARS-CoV-2 system has 100% sensitivity (≥ 14 days after a positive polymerase chain reaction [PCR] assay) and 99.8% specificity (https://www.fda.gov/medical-devices/coronavirus-disease-2019-covid-19-emergency-use-authorizations-medical-devices/eua-authorized-serology-test-performance). The results are reported as numeric values in the form of a cutoff index (COI; signal sample/cutoff) with qualitative results reactive (COI ≥ 1.0; positive). The analytical and clinical performance of the assay have been evaluated and are described elsewhere ^6^.

### Statistical analysis

Regarding the sample size, we had no prior information on the prevalence in the frontline HCWs of central Tokyo during the observation period. Since the prevalence of COVID-19 is low in Japan (a total of 43,815 confirmed cases and 1552 death till August 2020 according to the WHO situation report 200) (https://www.who.int/docs/default-source/coronaviruse/situation-reports/20200807-covid-19-sitrep-200.pdf?sfvrsn=2799bc0f_2), we also expected the seroprevalence among HCWs to be low. Therefore, we first used ‘Zero Patient’ method to estimate necessary sample size^7^. According to the theory, in a sample size of n with zero prevalence, the 95% upper bound CI for prevalence *P* can be calculated from the following Eq. ^7^: α = (1 – *P*)^n^, where $\mathrm{\alpha }$ is the probability of rejecting. With a low prevalence (*P* = 0.00075) and a significant level of 0.05 (α = 0.05), n is calculated to be 4000, which was similar to our sample size. Bootstrap procedure was used to estimate confidence bounds for the prevalence. We used the basic percentile of the bootstrap distribution to construct confidence intervals, repeating the steps above for 10,000 bootstrap samples^8^. To adjust for the test performance characteristics, combinations of sensitivity and specificity (80% sensitivity and 100% specificity, and 90% sensitivity and 100% specificity) were applied upon an assumption of the exact binomial distribution. Second, we also used the Bayesian estimation, which is used for probability distributions to reflect uncertainty of parameters in a model. Because SARS-CoV-2 lateral flow assays are new, we used several different combinations of sensitivity and specificity in test performance. We used estimated test sensitivity and specificity based on the B test kit sheet^7,8^. For our analyses, we set informative prior distributions for the sensitivity and specificities of 99%. We constructed 95% credible intervals (95% CrI) representing the values between which there is a 95% probability of containing the parameter of interest, given the data and the prior information used^9^.

Statistical analyses were performed using Epitools developed by AUSVET, SAS 9.4 (SAS Institute Inc., Cary, NC) and Stat Flex for Windows (ver. 6.0; Artech, Osaka, Japan). Chi-square tests or Fisher’s exact tests were used to evaluate the significance of differences in proportions between groups. The Mann–Whitney U test was used to compare the COI between the symptomatic and asymptomatic HCWs with SARS-CoV-2 antibodies. A two-tailed *p* value of < 0.05 was considered statistically significant.

## Results

A total of 4147 HCWs at the Juntendo University Hospital and employees of the Juntendo University Graduate School of Medicine participated in this study. Table 1 shows the age and sex composition of the study participants. The mean age was 36.8 years (standard deviation 12.0) years. Of all participants, 36.1% were male and 63.9% were female.

**Table 1:** Baseline characteristics of the study participants.

Characteristics	Participants (n = 4147)
**Age, years**	
20-29	1461 (35.2%)
30-39	1245 (30.0%)
40-49	776 (18.7%)
50-59	424 (10.2%)
60-69	193 (4.7%)
70-	48 (1.2%)
**Sex**	
Male	1498 (36.1%)
Female	2649 (63.9%)

Data are presented as n or n (%).

Of the 4147 participants, 14 showed positive results for SARS-CoV-2 antibody, yielding a crude seroprevalence rate of 0.34% (exact binomial 95% confidence interval [CI] 0.18–0.57, Table 2). To evaluate accuracy of the crude seroprevalence, we calculated estimated prevalence using bootstrap method^8^, yielding the estimate seroprevalence of 0.337% (bootstrap 95% confidence interval [CI] 0.191–0.460). When test sensitivity of 90% and specificity of 100% were used, the estimated seroprevalence was 0.38% (exact binomial 95% confidence interval [CI] 0.22—0.63). When sensitivity of 80% and specificity of 100% were used, the estimated seroprevalence was 0.42% (exact binomial 95% confidence interval [CI] 0.25—0.71). In addition, Bayesian simulation showed the estimated seroprevalence was 0.20% (95% credible interval [CrI] 0–0.6). There was no significant difference in seroprevalence between male (0.27%, 4/1498) and female (0.38%, 10/2649) participants (*p* = 0.556), and no significant association with age: 20–29 years (0.27%, 4/1461), 30–39 years (0.40%, 5/1245), 40–49 years (0.39%, 3/776), 50–59 years (0.24%, 1/424), 60–69 years (0.52%, 1/193), and over 70 years (0%, 0/48) (*p* = 0.891).

**Table 2:** Antibody seroprevalence among healthcare workers according to the risk of exposure and professional category.

Exposure risk	Participants	Antibody positive
**High-risk exposure (HR)**		
Medical doctors	(n = 1111)	5 (0.45%)
Nurses	(n = 1308)	3 (0.23%)
**Medium-risk exposure (MR)**		
Laboratory personnel	(n = 236)	0 (0.00%)
Paramedics	(n = 314)	2 (0.64%)
**Low-risk exposure (LR)**		
Administrative staff	(n = 510)	3 (0.59%)
Researchers	(n = 632)	1 (0.16%)
Other	(n = 36)	0 (0.00%)
All	(n = 4,147)	14 (0.34%)

Data are presented as n or n (%).

The seroprevalence rate for SARS-CoV-2 in HCWs in different job categories is shown in Table 2. The seroprevalence was highest in paramedics and lowest in laboratory workers. There was no significant difference in seroprevalence among frontline HCWs (HR 0.33%, MR 0.36%, and LR 0.34%). In addition, none of the HCWs working in dedicated COVID-19 outpatient clinics and wards, including medical doctors (n = 244) and nurses (n = 224), had positive results for SARS-CoV-2 antibody.

**Figure 1:**
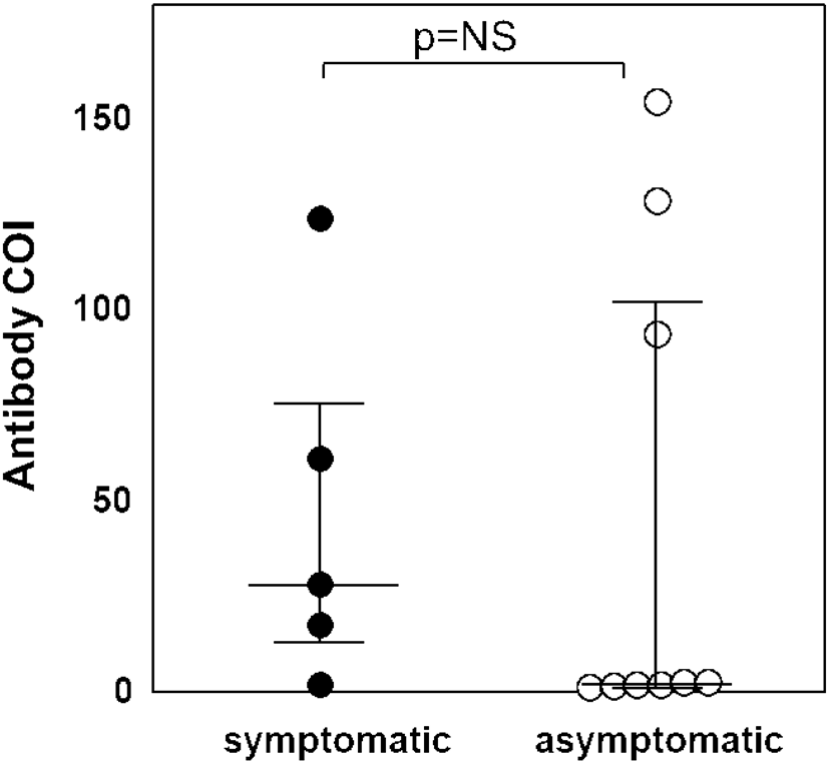
Comparison of severe acute respiratory syndrome coronavirus 2 (SARS-CoV-2) antibody levels among symptomatic and asymptomatic healthcare workers with antibodies against SARS-CoV-2. Antibody levels were quantified using the anti-SARS-CoV-2 antibody index (cutoff index, COI) in participants who had a positive result in the Elecsys Anti-SARS-CoV-2 electrochemiluminescence immunoassay. A COI of ≥ 1.0 was considered positive. Median values of the COI with lower quartile and upper quartile are shown according to symptom status. The difference in the median antibody level between the symptomatic and asymptomatic groups was not significant.

Transmission can occur from a patient to an HCW and between HCWs. We therefore interviewed all the seropositive participants about these interactions and confirmed that one of the paramedics was infected at work. Among the 14 seropositive participants, only five (36%) had been diagnosed with SARS-CoV-2 infection based on PCR results and had experienced some COVID-19 symptoms (Table 3). Of note, two participants who had been diagnosed with SARS-CoV-2 infection based on PCR results had negative results for SARS-CoV-2 antibodies. The numeric values of the cutoff index (COI) for the SARS-CoV-2 antibody were divided into a group showing high values (≥ 10) and a group showing low values near the cutoff (1–10). Of the five participants who had been diagnosed with symptomatic COVID-19, confirmed by PCR results, only 1 (20%) showed a low COI, and among nine asymptomatic participants, 67% (6/9) were antibody-positive with a low COI (Fig. 1). The median COI of the symptomatic and asymptomatic groups was 28.2 (interquartile range, IQR 13.5–76.7) and 2.5 (IQR 1.9–102.8), respectively. However, this difference in antibody levels between symptomatic and asymptomatic groups was not significant because of the small number of positive participants, and the large variation in antibody titers.

**Table 3:** Line listing of the clinical and demographic characteristics and the antibody levels of healthcare workers who tested positive for SARS-CoV-2 antibodies.

Participant	Age, years	Sex	Antibody COI	Symptomatic/asymptomatic	COVID-19 diagnosis *	Symptoms
#1 Medical Doctor	49	F	28.20	Symptomatic	+ (87 days)	Dysgeusia
#2 Medical Doctor	28	F	155.00	Asymptomatic	–	
#3 Medical Doctor	40	F	2.58	Asymptomatic	–	
#4 Medical Doctor	38	M	2.07	Asymptomatic	–	
#5 Medical Doctor	37	M	1.46	Asymptomatic	–	
#6 Nurses	29	F	129.00	Asymptomatic	–	
#7 Nurses	25	F	2.53	Asymptomatic	–	
#8 Nurses	33	M	1.86	Symptomatic	+ (46 days)	Fever, sore throat
#9 Paramedics	27	F	17.40	Symptomatic	+ (87 days)	Dysgeusia, olfactory disorder, fever, nasal discharge
#10 Paramedics	51	F	94.00	Asymptomatic	–	
#11 Administrative staff	35	F	60.90	Symptomatic	+ (41 days)	Olfactory disorder, fever, diarrhea, fatigue
#12 Administrative staff	63	F	124.00	Symptomatic	+ (91 days)	Cough, chest pain, fatigue
#13 Administrative staff	45	F	1.10	Asymptomatic	–	
#14 Researchers	34	M	2.01	Asymptomatic	–	

*COVID-19 diagnosis was made using RT-PCR (days from diagnosis date to antibody detection date).

COVID-19: coronavirus disease; SARS-CoV-2: severe acute respiratory syndrome coronavirus 2; RT-PCR: reverse transcription-polymerase chain reaction; M: male; F: female; COI: cutoff index.

## Discussion

This study demonstrated a low seroprevalence rate of SARS-CoV-2 antibodies in HCWs working at a frontline hospital in the Tokyo area after the first wave of the COVID-19 pandemic in Japan between March and May 2020.

The lower prevalence of SARS-CoV-2 antibodies observed in HCWs reflects the lower circulation of SARS-CoV-2 in Japan than in some other countries where more cases were reported. A high incidence of infection has been reported among COVID-19 frontline medical staff in China^10^ and a higher prevalence of SARS-CoV-2 antibodies has been reported in HCWs in England, Belgium, and the Unite d States (6%^11^, 6.4%^12^, and 33%^13^, respectively).

The Japanese government survey on the SARS-CoV-2 antibody-positive rate from June 1 to 7, 2020, targeting local residents in Tokyo (n = 1971), and other representative cities in Osaka (n = 2970), and Miyagi (n = 3009) prefectures using the Elecsys Anti-SARS-CoV-2 immunoassay, demonstrated antibody prevalence rates of 0.30%, 0.34%, and 0.23%, respectively^14^. Our survey, which was conducted with the same assay system, revealed no significant difference in the prevalence of seropositivity between frontline HCWs and the general public in the Tokyo area.

We further observed no significant difference in the seroprevalence of SARS-CoV-2 antibodies between frontline HCWs and those working in other settings, which is inconsistent with the findings of previous reports that showed a higher seroprevalence in HCWs with higher exposure to COVID-19^15^. These results may be partly due to the limited size of the pandemic in Japan, with ample access to protective equipment and sufficient time to prepare to prevent transmission. During the COVID-19 pandemic, the provision of adequate health care to patients is highly reliant on HCWs who are safe and well protected^16^. Along with continuing to observe the recommend standard protective precautions per CDC guidelines, determination of the seroprevalence of SARS-CoV-2 antibodies in HCWs helps to estimate the safety of their work environment.

Of the seropositive HCWs, 64% reported an absence of any signs or symptoms in the previous three months, which is consistent with the findings of previous studies that showed that almost 90% of infections among HCWs in the United States were asymptomatic^17,18^. Our results show that some individuals with SARS-CoV-2 infection develop antibodies without displaying signs of disease.

Similar to our data, several studies reported low seroprevalences among HCWs^19–21^. For example, in Greek where the prevalence of COVID-19 was only 0.12%, the overall seroprevalence in 1495 HCWs in two hospitals was 1.26% (95% CI 0.43, 3.26)^19^. In Italy where the infectious rate was relatively high, the prevalence in 115 HCWs was 3.4%^20^. Among 2924 HCWs in California, U.S.A., the observed prevalence was 1.06%, and the adjusted seroprevalence for test sensitivity and specificity was 1.06%^21^. However, the low seroprevalence rate in this study raises the risk of detecting false-positive results, due to a low positive predictive value. The fact that most study participants who were designated "asymptomatic" had very low COI values raises the concern that some false-positive results may have been reported. The high rate of asymptomatic infections in this study may, in part, be due to false-positive antibody tests.

The nature and strength of innate and adaptive immune responses in such cases require further investigation. A better understanding would be obtained by ongoing surveillance. Systematic surveillance of the proportion of seropositive HCWs is an important indicator of the spread of SARS-CoV-2 and is useful for assessing the effectiveness of infection prevention in the medical setting. To the best of our knowledge, this study is one of the largest systematic screening study of SARS-CoV-2 seroprevalence in HCWs in Japan.

The strengths of this study include the following: (1) the study scope and screening for SARS-CoV-2 antibodies have aided in the detection of all cases of SARS-CoV-2 infection to date; (2) the study had high participation of HCWs who submitted the mandatory web-based questionnaire; and (3) participants were not selected for screening on the basis of the presence of symptoms. Limitations include the following: (1) the study is a single survey conducted in a single institution that cannot be considered representative of the entire healthcare workforce in the Tokyo area; (2) The Elecsys Anti-SARS-CoV-2 test utilized in this study is a qualitative test expressing qualitative statements (COI, reactive/non-reactive) and is not possible to accurately identify the high and low values that attribute an exact clinical or prognostic meaning; and (3) The relationship between currently measured antibodies and neutralizing activity against SARS-CoV-2 was not evaluated. Therefore, we have planned to perform a follow-up survey for five more years to better understand the duration of immune response and protection from reinfection by measuring neutralizing antibodies through a confirmation assay (e.g., plaque reduction neutralization test) and quantitative tests. The WHO emphasizes the importance of repeated cross-sectional analyses of seroprevalence as a COVID-19 tracking system that might be able to capture the incidence of exposure in both symptomatic and asymptomatic individuals.

This study has limitations. The primary limitation is sample selection bias since the study subjects were from a single university hospital. Second, the prevalence was estimated in a specific COVID-19 antibody test whose true sensitivity and specificity were uncertain. Therefore, we simulated the prevalence by Bayesian method using various combinations of sensitivities and specificities. However, true accuracy for various COVID-19 tests including RT-qPCR remains unknown^22,23^.

In conclusion, this survey provides an insight into the extent of infection and immune status in the Japanese HCW population (i.e., Juntendo University Hospital, Tokyo area); (1) overall low seroprevalence of SARS-CoV-2 antibodies, (2) the frontline HCWs working in hospitals and HCWs working on a dedicated COVID-19 ward showed no significant difference in seroprevalence when compared with other HCWs with low exposure risk, (3) more than half of seropositive HCWs had no symptoms attributable to SARS-CoV-2, which strengthens the hypothesis that “asymptomatic” cases involve the development of a specific immune response against SARS-CoV-2.

Our results have relevance for public health policy to control virus spread in a region with a low-intensity COVID-19.

### Informed consent and ethical approval

Research involving human subjects complied with all relevant national regulations and institutional policies and is in accordance with the tenets of the Helsinki Declaration (as revised in 2013). Informed consent was obtained in accordance with the Juntendo University Hospital IRB-approved protocol (IRB #20–089).
